# Identification of defense related gene families and their response against powdery and downy mildew infections in *Vitis vinifera*

**DOI:** 10.1186/s12864-021-08081-4

**Published:** 2021-10-30

**Authors:** Neetu Goyal, Garima Bhatia, Naina Garewal, Anuradha Upadhyay, Kashmir Singh

**Affiliations:** 1 Department of Biotechnology, Panjab University, BMS Block I, Sector 25, Chandigarh, -160014 India; 2 National Research Centre for Grapes, Solapur Road, Pune, Maharashtra 412 307 India

**Keywords:** Grapevine, Mildew resistance, Defense related genes, Host-pathogen interactions, Biotic stress

## Abstract

**Background:**

Grapevine (*Vitis vinifera)* productivity has been severely affected by various bacterial, viral and fungal diseases worldwide. When a plant is infected with the pathogen, various defense mechanisms are subsequently activated in plants at various molecular levels. Thus, for substantiating the disease control in an eco-friendly way, it is essential to understand the molecular mechanisms governing pathogen resistance in grapes.

**Results:**

In our study, we performed genome-wide identification of various defensive genes expressed during powdery mildew (PM) and downy mildew (DM) infections in grapevine. Consequently, we identified 6, 21, 2, 5, 3 and 48 genes of *Enhanced Disease Susceptibility 1 (EDS1), Non-Race-specific Disease Resistance (NDR1), Phytoalexin deficient 4 (PAD4), Nonexpressor of PR Gene (NPR), Required for Mla-specified resistance (RAR)* and *Pathogenesis Related (PR)*, respectively, in the grapevine genome. The phylogenetic study revealed that *V. vinifera* defensive genes are evolutionarily related to *Arabidopsis thaliana.* Differential expression analysis resulted in identification of 2, 4, 7, 2, 4, 1 and 7 differentially expressed *Nucleotide-binding leucine rich repeat receptor* (*NLR), EDS1, NDR1, PAD4, NPR, RAR1* and *PR* respectively against PM infections and 28, 2, 5, 4, 1 and 19 differentially expressed *NLR, EDS1, NDR1, NPR, RAR1* and *PR* respectively against DM infections in *V. vinifera.* The co-expression study showed the occurrence of closely correlated defensive genes that were expressed during PM and DM stress conditions.

**Conclusion:**

The PM and DM responsive defensive genes found in this study can be characterized in future for impelling studies relaying fungal and oomycete resistance in plants, and the functionally validated genes would then be available for conducting *in-planta* transgenic gene expression studies for grapes.

**Supplementary Information:**

The online version contains supplementary material available at 10.1186/s12864-021-08081-4.

## Background

Grapevine is one of the most commercially valuable fruit crops grown around the world. Nevertheless, its growth is significantly impacted by climate in the mass-producing areas, which allows for the development of various pathogenic diseases. Powdery mildew (PM) and downy mildew (DM) are two of those destructive diseases that propagate in the areas with colder temperatures, and higher relative humidity infecting grapevine [1]. The causal agent of PM is *Erysiphe necator*, an ascomycete fungus, while DM is caused by an oomycete, *Plasmopara viticola.* The epidemic of these diseases acutely diminishes both the productivity as well as quality by infecting all the green tissues in grapevine. As majority of the cultivated varieties belong to *Vitis vinifera*, which is susceptible to both PM and DM, it is vital to understand the molecular processes involved in grapevine resistance to powdery and downy mildew infection in order to manage these diseases in an eco-friendly manner. Under abiotic and biotic stress environments, different defensive mechanisms are triggered in plants as part of their innate immune response to protect themselves. Essentially, three main steps are involved in host defense against pathogenic attack, i.e. pathogen identification, signal transduction and gene induction; contributing to the production of molecules with antimicrobial properties. Once the emergence of PM and DM infections establishes in grapes, under optimal environmental conditions, the conidiospores of *Erysiphe necator* and *Plasmopara viticola* germinate on the grapevine leaf surface. This resulted in initial association of pathogen-associated molecular patterns (PAMPs) and grapevine defense molecules known as pattern recognition receptors (PRRs), thereby activating the first defense layer i.e. Pathogen-triggered immunity (PTI) [2]. However, the activated defense is not strong enough to deter the pathogenic augmentation in plants. In addition, due to evolution, certain pathogenic isolates have adapted to producing effector proteins in response, that are delivered within the cell, hence the PTI gets suppressed [3, 4]. During that time, another defense mechanism known as effector-triggered immunity (ETI) is activated in plants that triggers the expression of intracellular resistance (R) proteins often leading to apoptosis of the infected cell, thus, blocking any further pathogenic proliferation [5–9].

During plant immune response, numerous signal transduction pathways are triggered which lead to the host plant transcriptome re-programming and activation of the defense-responsive genes. Activated nucleotide binding sites leucine rich repeats (NLRs) trigger a cascade of intracellular immune responses, including an increase in reactive oxygen species (ROS) production, mitogen-activated protein kinase (MAPK) cascades, calcium spikes, phytohormone production, transcriptional reprogramming etc. [10]. Various plant hormones function as key players in activating the plant immune signaling network downstream the PTI or ETI induction [11–14]. Most often, resistance to biotrophic and hemibiotrophic pathogens is driven by salicylic acid (SA) signaling, while resistance to necrotrophic pathogens is mediated by jasmonate (JA) and ethylene (ET) signaling [15]. Since *E. necator* and *P. viticola* are both biotrophic pathogens, therefore, the defense mechanism triggered in the plant in response to infection with powdery and downy mildew might be modulated by SA signaling pathway. Once the SA signaling is initiated at the infection site, a similar response is triggered in undamaged plant tissues to provide protection against further pathogen invasion. This broad-spectrum long lasting resistance response is known as systemic acquired resistance (SAR) [16].

In the present research, we have focused on *R-gene* mediated defense signaling through SA accumulation. Once PAMPs or pathogen effectors are recognized in PTI and ETI, SA production is activated [17]. The *R-gene* mediated resistance has been previously documented to be triggered by 2 main proteins i.e. Enhanced Disease Susceptibility 1 (EDS1) and Non-Race-specific Disease Resistance (NDR1), both interacting with different types of resistance (R) proteins. EDS1 interacts with Toll/interleukin-1 receptor (TNL) type R proteins and NDR1 interacts with Coiled coil (CNL) type R proteins [18–20]. Recent study on Arabidopsis have demonstrated that any perturbation in the interactions between EDS1, NDR1 and R proteins can affect the activation of SA signaling pathway, thereby indicating the importance of these two main plant defense regulators [21–24]. Further, EDS1 forms heterodimers with Phytoalexin deficient 4 (PAD4) and Senescence-associated gene 101(SAG101), two proteins with which it shares sequence homology [25, 26]. These three proteins’ N-terminal domains allowed EP domains at C-terminal to bind and form interaction surfaces on the heterodimer [27].

Several other major molecules of SA mediated defense signaling includes *avrPphB susceptible 2* (*PBS2)*, *required for Mla-specified resistance* (*RAR1), Nonexpressor of PR Gene* (comprising of *NPR1, NPR2, NPR3* and *NPR4*). RAR1, a chaperone protein is an important member of NLR mediated ETI utilized by both TIR-NLR and CC-type NLR [28]. The NPR class is a pivotal SA-mediated signal transduction regulator that is triggered after accumulation and subsequently binding of SA [29]. NPR1, NPR2, NPR3, and NPR4 are among the members of this protein family. According to the literature survey, NPR1 and NPR2 positively regulate SAR, while NPR3 and NPR4 regulate SAR negatively [30, 31]. The SA binds to NPR’s ankyrin-repeat domain, liberating it from BTB/POZ domain’s auto-inhibition. The C-terminal domain then associates with transcription factors (TFs) of WRKY and TGA class in nucleus, and further it binds with promoter sequences of *Pathogenesis Related (PR)* genes [32–34]. Recently, structural analyses of SA detection by several NPR proteins have been published, which aids in better understanding of mechanism of SA recognition by NPR proteins [35]. The ultimate players of plant defense mediated through SA are PR proteins that were described firstly in tobacco leaves infected with the tobacco mosaic virus [36]. Since then, these proteins have been identified in many monocot and dicot plants. Although these genes are wide-ranging, many of them are encoding for antimicrobial proteins [37]. PR-1 is a well-studied PR gene that is often used as a strong indicator of SA-mediated gene expression.

The importance of the role of SA or its derivatives in triggering defense-responsive proteins is well stated in literature. For instance, the plant susceptibility to pathogen increases when the plant becomes deficient in SA synthesis or accumulation [38]. It is evident in literature through experiments performed in plants like Arabidopsis that *eds5* and *eds16* mutants that are lacking SA accumulation are compromised in some *R-gene* pathways as well as SAR and basal resistance [38]. Various PR proteins that are known to induce SAR, get expressed when the plant is treated with SA or its derivatives [39] . This suggested the importance of SA in providing immunity to plants whenever the plant gets pathogenic infection. Previously, we identified 386 *NLR* genes in the grape genome, of which 63 such genes were found to be responsive to PM stress [40]. In the present study, we have carried out the genome-wide identification of various defense-responsive gene families (*EDS1, NDR1, PAD4, NPR, RAR1* and *PR*). The evolutionary relatedness of identified gene families is studied with model plant *Arabidopsis thaliana.* The differential expression studies are conducted based on the PM and DM-responsive RNA seq data. As a result, we have identified various PM and DM-responsive defensive genes of above mentioned gene families (*EDS1, NDR1, PAD4, NPR, RAR1 and PR*) along with their co-expression to identify highly correlative defensive genes responsive to both PM and DM infections. The different genes obtained through co-expression analysis are functionally characterized by the Blast2GO results. Additionally, various *cis*-acting regulatory elements (CARE) for the identified genes have also been predicted against the PM and DM stresses.

## Results

### Genome-wide identification of various defense-responsive gene families in *Vitis vinifera*

The genome-wide identification of various classes of defensive genes in *V. vinifera*, namely *EDS1, NDR1, PAD4, NPR, RAR1* and *PR* in was conducted by running standalone BLASTP between protein coding sequences of *V. vinifera* and defensive gene families. For analysis, we collected 38,120 protein coding sequences of *V. vinifera* from the NCBI genome database. Next, we retrieved 41, 127, 17, 34, 9 and 92 coding sequences of EDS1, NDR1, PAD4, NPR, RAR1 and PR proteins respectively from *A. thaliana, A. lyrata, O. sativa, P. trichocarpa* and *V. vinifera* using NCBI. The sequence alignments were done and consequently, 27, 47, 29, 928, 3 and 148 putative gene hits from *EDS1, NDR1, PAD4, NPR, RAR1* and *PR* gene families were detected in *V. vinifera*. The confirmation of the presence of respective domains was done through BLASTP analysis of putative hits against the non-redundant (NR) database. Finally, 6, 21, 2, 5, 3 and 48 such sequences were obtained that were considered as EDS1, NDR1, PAD4, NPR, RAR and PR proteins after validation through bioinformatics tools and databases such as Genoscope, CDD, Pfam, InterProScan and SMART. The information regarding protein IDs and transcript IDs of all gene families is given in Additional file 1.

The identified genes were characterized in silico on the basis of gene structure, motif presence, chromosomal locations and physicochemical properties. The gene structure analysis was performed by using SPLIGN tool which provided valuable information about gene arrangement in terms of exons and introns. The results depicted that the number of introns in the *EDS1, NDR1, PAD4, NPR, RAR1* and *PR* gene families ranged from 1 to 2, 1–2, 1–3, 1–4, 1–4, 1–4 and 1–2 respectively (Additional file 2). Further, on the basis of the localization of an intron with respect to reading frame of gene, three intron phases were depicted. All *EDS1* gene sequences were present in phase 0; *NDR1* in phase 0 and phase 1; *PAD4* in phase 1 and phase 2; *NPR, RAR1* and *PR* gene sequences in all the three intron phases respectively (Additional file 2). Next, the protein sequences were assessed with ‘MEME’ server to identify conserved motifs. A total of 16 conserved motifs were identified in the studied gene families (Additional file 2). Their final verification for the presence of desired motif was done through Pfam server and InterProScan. Conserved domains identified were Lipase 3 and EDS1 EP in EDS1; LEA2 domain in NDR1; EDS1 EP in PAD4; Ank2, Ank5, NPR1_like_C; NPR1_interact in NPR; CHORD domain in RAR1; CAP, Thaumatin, Glycohydro 17, Glycohydro 18, Glycohydro 19, Betv1, Barwin and Chitin_bind_1 in different classes of PR coding sequences. The block diagrams and the logos of the conserved motifs are shown in additional file 2.The identified genes were found to be distributed widely in the grape genome. All *EDS1, PAD4* and *RAR1* genes were located on single chromosome i.e. 17, 7 and 16; *NPR* genes were located on chromosomes 7, 8, 10 and 11; *NDR1* and *PR* genes were unevenly distributed at several chromosomes. However, no gene sequences were identified on chromosome number 1 and 12 in any gene family (Additional file 2). The various parameters computed to determine physicochemical properties included amino acid length, molecular weight, isoelectric point (pI) and instability index (Additional file 1). The range of amino acid length for the studied proteins is 112–629 and the molecular weight range is between 12 and 71.3 kDa. Based on pI prediction, it was depicted that EDS1 proteins were acidic, basic and neutral in nature; all the NDR1 proteins were basic in nature; PAD4 and NPR proteins were acidic; RAR1 proteins were neutral and basic in nature; PR were mostly acidic and some are basic in nature. The results of instability index prediction suggested that most of the proteins identified were unstable under in-vitro conditions with the exception of some NDR1 and PR proteins of PR1, PR2, PR10.1, PR10.7 families that were found to be present in stable state.

### Phylogenetic analysis

**Fig. 1:**
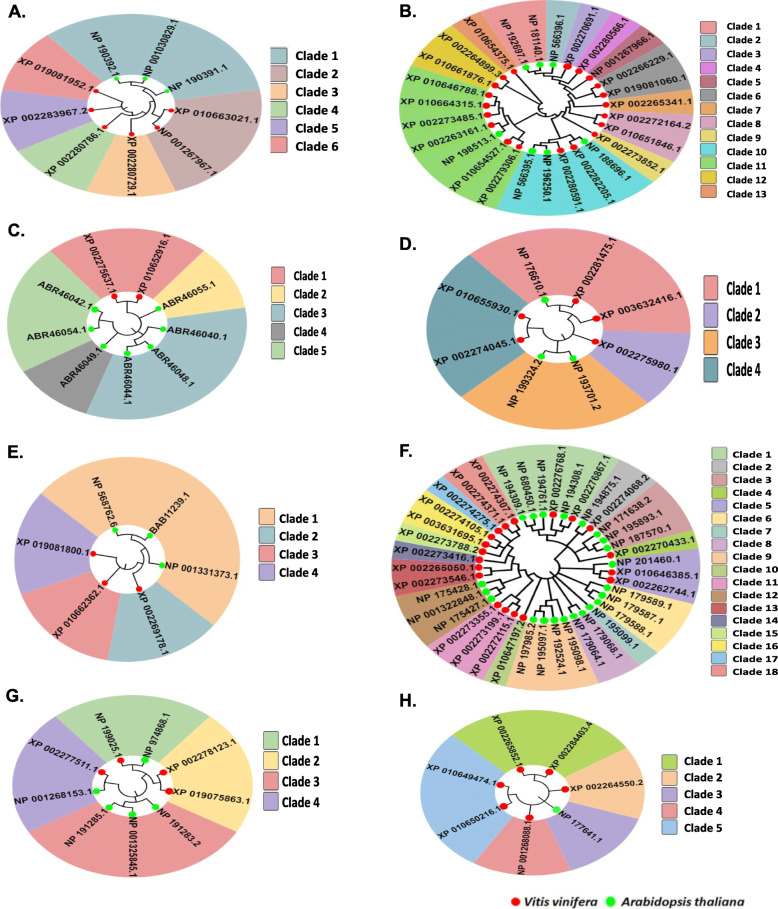
Phylogenetic trees of defense responsive proteins of *V. vinifera* and *A. thaliana* A.) EDS1, B.) NDR1, C.) PAD4, D.) NPR, E.) RAR1, F.) PR1, G.) PR2, H.) PR5. Visualization of phylogenetic trees was done through iTOL. The colour of the labels indicate different clades, while those of leaf nodes depict the species

The evolutionary history of *EDS1, NDR1, PAD4, NPR, RAR1* and *PR* genes was traced by conducting their phylogenetic analysis with model plant *A. thaliana.* Full length amino acid sequences of all genes families were used to perform multiple sequence alignment and subsequently, phylogenetic trees were constructed which were classified into different phylogenetic clades (Fig. 1). The analysis depicted that all the studied genes families of *V. vinifera* and *A. thaliana* were closely related. The phylogenetic tree of EDS1 proteins was classified into six clades, with clades II-VI comprising *V. vinifera* proteins exclusively and clade I carrying *A. thaliana* proteins. The NDR1 proteins from both plants were divided into 13 clades, with clades I, II, X and XI including primarily *A. thaliana* proteins and the remaining clades containing *V. vinifera* proteins. The PAD4, NPR, and RAR1 proteins were classified into five, four, and four clades, respectively, and the majority of the protein members of both plants were found in separate clades. Likewise, we constructed distinct phylogenetic trees for three different PR protein classes, namely PR1, PR2, and PR5. The results showed that PR1 proteins from both plants were classified into 18 clades, PR2 proteins were classified into four clades and PR3 proteins were grouped into five clades. Overall, the analysis showed that majority of the members of studied gene families from both plants were evolutionary conserved, hence clustered together and placed in distinct clades (Fig. 1).

### Identification of PM and DM-responsive defensive genes in *V. vinifera*

**Fig. 2:**
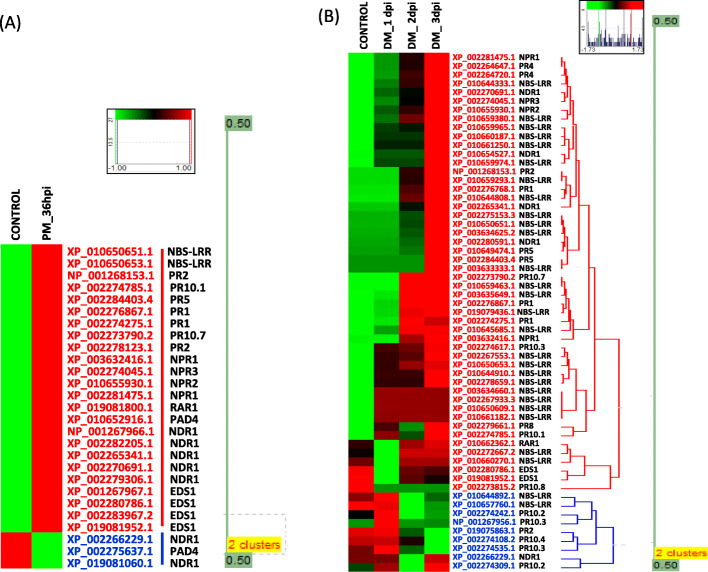
Heat maps illustrating the differential expression patterns for defensive genes in *Vitis vinfera.* (a) PM-responsive defensive genes at time points of 0 h and 36 hpi. The gradient value of color mapping ranges from − 1 to + 1 (b) DM-responsive defensive genes at time points of 0 h, 1 dpi, 2 dpi and 3 dpi. The gradient value ranges from − 1.73 to + 1.73. Green color indicates declined expression level, red color indicates elevated expression levels, black color indicates no expression. PM and DM defensive genes are clustered in two groups indicated by gene names with blue and red color

To better understand the *R-gene* mediated molecular mechanism of powdery and downy mildew resistance in grapevine, the identification of PM and DM-responsive defensive gene families (*NLR, EDS1, NDR1, PAD4, NPR, RAR1* and *PAD4*) and transcription factors (TF) was done by conducting the differential gene expression analysis. We reported the occurrence of 386 *NLR* gene sequences in the grape genome in our previous research, including 63 such sequences that were sensitive to PM stress [40]. In this study, we found 2 new highly expressing PM-responsive and 28 DM-responsive *NLR* genes. Additionally, 4 4, 7, 2, 4, 1 and 7 *EDS1, NDR1, PAD4, NPR, RAR1* and *PR* genes expressing differentially in reaction to PM infection stress and 2, 5, 4, 1 and 19 differentially expressed DM-responsive defensive genes (*EDS1, NDR1, NPR, RAR1* and *PR*) were identified (Fig. 2)*.* The differentially expressed genes were represented as heat maps that depicted the expression fold change. During analysis, it was observed that for the triplicates of each condition; the TPM values obtained were nearly same. Therefore, we took the cumulative mean of these values for every triplicate during heat map representation.

The TPM findings of *NLR* genes showed that most of the *NLR* genes were up-regulated in later stages of pathogen infection relative to initial phase during both PM and DM-infection (Fig. 2). Analysis of *EDS1* and *NDR1* gene families showed that all *EDS1* and 5 *NDR1* genes were up-regulated during PM-infection relative to control condition (Fig. 2A). Likewise, during DM-infection, all *EDS1* and 4 *NDR1* genes were up-regulated at 3 dpi (Fig. 2B). The analysis of *PAD4* gene family revealed that 2 *PAD4* genes were detected in PM infected samples, one of which was up-regulated (XP_010652916.1) while the other one was down regulated (XP_002275637.1) during PM infection as compared to control conditions (Fig. 2A). No differentially expressed *PAD4* genes were identified in DM-infected sample. We found 1 PM-responsive and 1 DM-responsive differentially expressed transcripts encoding *RAR1* in both PM and DM infection and both transcripts demonstrated up-regulation during pathogenic stress (Fig. 2). The DEA of NPR proteins indicated that 4 PM-responsive and 4 DM-responsive *NPR* (comprised of *NPR1* and *NPR2*) genes were expressed differentially during infection and all *NPR* genes were shown to be steadily up-regulated from initial to later stages of both powdery and downy mildew infection (Fig. 2).

Further, the DEA of 3 transcription factors (TFs) families i.e. TGA, WRKY and NAC was done as T.Fs are known to play a significant role in stimulating the *PR* genes expression during *R-gene* mediated defense signaling after interacting with *NPR* genes [32–34]. By using Plant Transcription Factor Database (TFDB), the total number of TGA, WRKY and NAC TFs retrieved was 47, 59 and 71. Their differential expression was conducted and consequently, 20 PM-responsive and 39 DM-responsive TFs were identified. Following that, we found 7 PM-responsive and 19 DM-responsive differentially expressed *PR* genes, with the majority of genes showing a significant surge in expression levels during infections.

Overall, we identified 27 PM-responsive and 59 DM-responsive differentially expressed defensive genes (*NLR, EDS1, NDR1, PAD4, NPR, RAR1 and PAD4*) in *V. vinifera* accessions used in the current study (Fig. 2). The findings indicated that most genes were up-regulated during PM and DM infection relative to control conditions.

### Co-expression analysis of PM and DM responsive defensive genes in *V. vinifera*

**Fig. 3:**
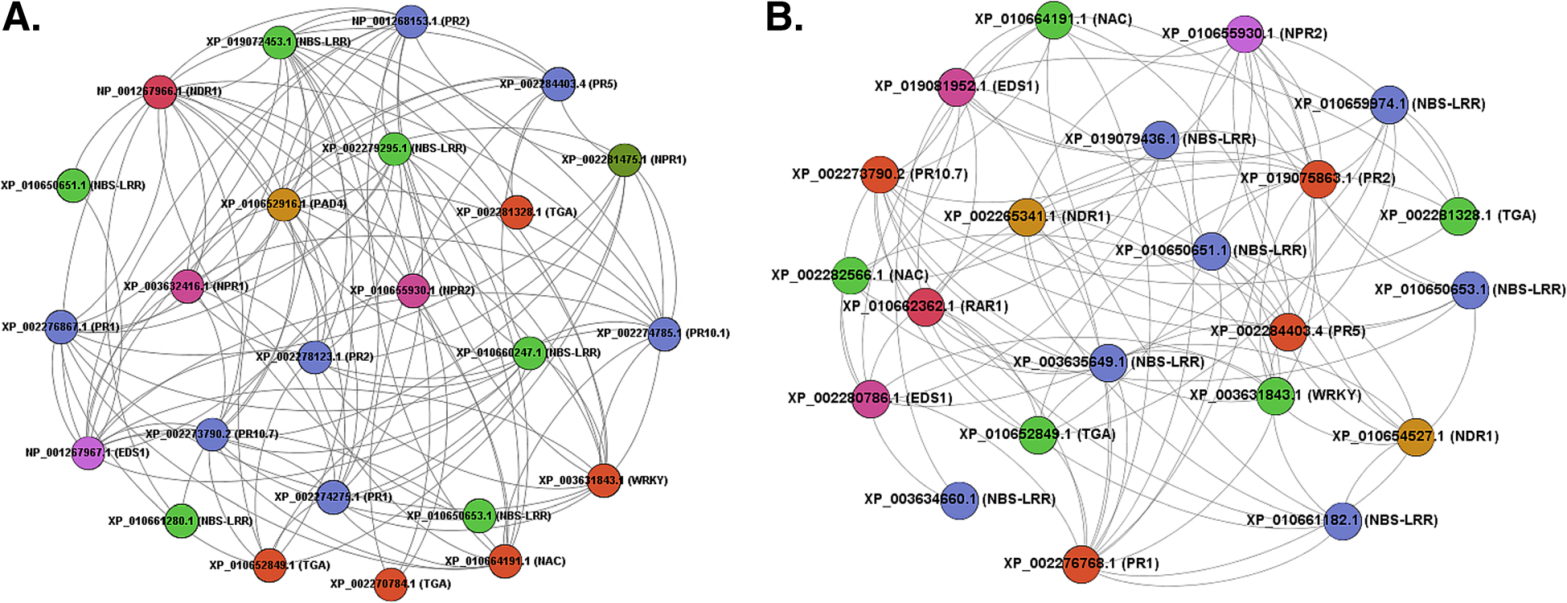
Co-expression analysis of defensive genes showing pairwise correlation of different protein families (NLR, EDS1, PAD4, NPR1, WRKY, TGA, PR) using  Gephi 0.91 software. (a) Co-expression amongst PM-responsive defensive genes visualized by cytoscape 3.7.1. (b) Co-expression amongst DM-responsive defensive genes was shown

As co-expression analysis effectively detects correlation between genes and provides an important information about which genes are expressing simultaneously, we conducted co-expression (CE) analysis of differentially expressed PM and DM-responsive defensive genes using the software tool CoExpress 1.5.2. Consequently, we screened some highly co-expressing genes in *V. vinifera* that could regulate the powdery and downy mildew infection through *R-gene* mediated signaling cascade (Fig. 3). During PM stress, 6 NLRs, 1 EDS1, 1 NDR1, 1 PAD4, 3 NPRs (comprised of 2 NPR1 and 1 NPR2), 5 TFs and 7 PRs co-expressed with 41, 15, 14, 15, 18, 36 and 73 other transcripts respectively, of defensive gene families as studied above. However, no RAR1 was found to be co-expressed with any transcript of defensive gene family during PM stress (Fig. 3A). Likewise, when differentially expressed DM-responsive genes were analyzed for co-expression, 7 NLRs, 2 EDS1, 2 NDR1, 1 RAR1, 1 NPR2, 5 TFs and 4 PRs co-expressed with 43, 19, 18, 13, 13, 38 and 44 other transcripts respectively (Fig. 3B). The exception in this case is PAD4 that was not found to be co-expressed with any defensive gene family transcript. Altogether, we screened 24 PM-responsive and 22 DM-responsive differentially expressed genes and TFs in *V. vinifera* accessions, which were found to be highly co-expressing amongst each other (Fig. 3).

### qPCR expression analysis

**Fig. 4:**
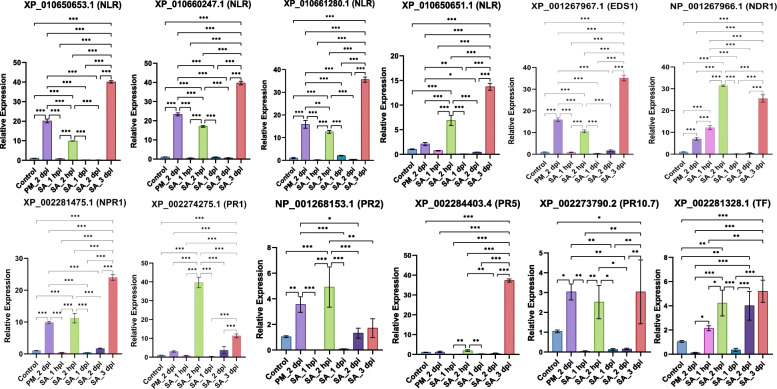
Relative expression levels of PM-responsive defensive genes during PM infection and salicylic acid (SA) treatment. The fold change was calculated relative to healthy leaf samples which were assigned a value of 1. Error bars indicate the standard deviation (SD) of means. Significant differences at *P* < 0.001, *P* < 0.01, *P* < 0.1 have been shown with 3 (***), 2 (*) and 1 (*) asterisks

**Fig. 5:**
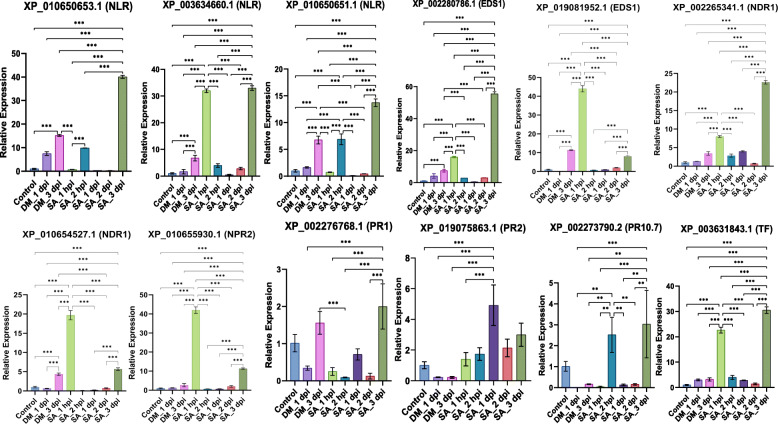
Relative expression levels of DM-responsive defensive genes during DM infection and salicylic acid (SA) treatment. The fold change was calculated relative to healthy leaf samples which were assigned a value of 1. Error bars indicate the standard deviation (SD) of means. Significant differences at P < 0.001, P < 0.01, P < 0.1 have been shown with 3 (***), 2 (*) and 1 (*) asterisks

On the basis of TPM and co-expression analysis, we picked 14 PM-responsive and 13 DM-responsive highly expressing defensive genes for real time expression analysis under various stress conditions (Fig. 4 and Fig. 5). The primer sequences, amplicon length and annealing temperature of PM and DM-responsive defensive genes are listed in Additional file 3.

For analysis, 3 different types of stresses were chosen i.e. PM stress, DM stress and salicylic acid (SA). In previous studies, it was seen that the exogenous application of SA or SA analogs induces SAR and restores resistance in numerous mutants compromised in signaling steps upstream of SA production [25, 41]. In the present study, as we are focusing on genes of *R-gene* mediated defense signaling which is known for having the involvement of SA signaling, therefore we studied its effects on resistance response against PM and DM infections. On the leaves of Thompson seedless variety, the SA was applied exogenously at 5 different time intervals i.e. 1 hpi, 2 hpi, 1 dpi, 2 dpi and 3 dpi to test the expression pattern changes of various defense-responsive genes. In addition, we also collected PM and DM infected leaf samples at various time intervals of 1 dpi for PM and 1 dpi and 3 dpi for DM infection and performed qPCR. The results of qPCR indicated that mostly the expression levels of PM-responsive defensive genes were higher during 2 dpi PM infection, 2 hpi and 3 dpi SA treatment (Fig. 4). At 1 hpi of SA treatment, the higher relative expression of only *NDR1* gene was observed compared to other gene families; after 1 hpi and upto 2 hpi of SA treatment, expression levels of all genes increased; however at 1 dpi SA treatment, all genes were down regulated compared to 2 hpi; then slight increase in expression level at 2 dpi; and at 3 dpi of SA treatment, maximum up-regulation was observed and all genes were up-regulated compared to 2 dpi. Overall, it was found that *PR* genes were showing the maximum expression at 2 hpi; all other gene classes (*NLR, EDS1, NDR1, NPR2*) were showing maximum expression at 3 dpi. Additionally, for PM-infected sample at 2 dpi, the relative expression levels for all genes were higher compared to control condition. Also, the expression levels for all gene families at 2 dpi PM-infection was comparable to that of SA treated samples at 2 hpi and 3 dpi (Fig. 4).

Likewise, the expression levels of DM-responsive defensive genes were higher mainly during late infection i.e. 3 dpi DM infection, 1 hpi and 3 dpi SA treatment (Fig. 5). At 1 hpi of SA treatment, the relative expression of all genes was higher; expression level decreased from 1 hpi to 2 hpi of SA treatment for all genes; however at 1 dpi of SA treatment, there was an increase in expression levels of some of the genes (*NLR, NDR1, PR2*); at 2 dpi, almost similar expression levels as that at 1 dpi was observed; however, at 3 dpi, all genes were up-regulated as compared to 2 dpi condition. At 1 dpi of DM infection, the relative expression levels of *NLR, NDR1, EDS1, NPR2* and *TGA* TFs coding genes was higher as compared to control. However, none of the *PR* coding genes were showing an up-regulation. At 3 dpi of DM infection, the expression levels of most of the genes increases as compared to 1 dpi condition. Additionally, at 3 dpi of DM infection, the slight increase in expression level of *PR1* gene was observed (Fig. 5). Overall, if we compare an expression levels between 1 dpi and 3 dpi of DM infection, expression levels of all genes increased from 1 dpi to 3 dpi DM infection except *PR2* gene that was down regulated and *PR1* gene that was up-regulated at 3 dpi DM infection. Moreover, if we compare 1 dpi and 3 dpi SA treatment condition and 1 dpi and 3 dpi DM infection; almost similar trend was followed from 1 dpi to 3 dpi for all genes.

### Functional annotation of PM and DM-responsive defensive genes

In order to recognize the diverse roles of proteins at the molecular level, we performed Blast2GO of highly interacting defensive genes found in the co-expression study. From this analysis, the depiction of biological process, cellular component and molecular function, the 3 key characteristics of genes was done. Various GO terms were assigned to gene sequences for determining the possible functions of PM and DM-responsive defensive genes. For both PM and DM-responsive genes, in the biological process category, we obtained maximum gene sequences that were stimulus responsive (GO:0050896). [Additional file 4A(a) and Additional file 4B(a)]. Likewise, the GO term annotations in cellular component category suggested that most of our gene sequences performed their respective functions in cell (GO:0005623), cell part (GO:0044464) and organelle (GO:0043226) [Additional file 4A(b) and Additional file 4B(b)]. The findings of molecular process category revealed that maximum number of sequenceswere involved in binding activity (GO:0005488)) [Additional file 4A(c) and Additional file 4B(c)]. Altogether, our results depicted that the PM and DM-responsive defensive genes found in our study may play an immense role in biological, cellular and molecular processes.

### Prediction of *cis*-acting regulatory elements in PM and DM-responsive defensive genes

**Fig. 6:**
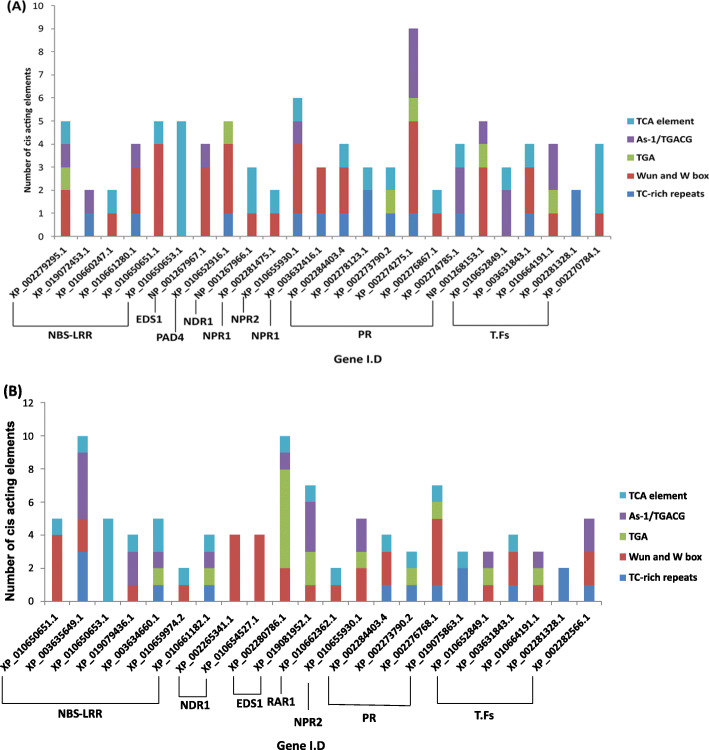
Assessment of promoter sequences of stress responsive defensive genes. (a) PM-stress responsive (b) DM-stress responsive *cis*-elements were shown in different colors. Each color represents different *cis*-element

Predictions of *cis*-regulatory elements in PM and DM-responsive defensive genes derived from a co-expression study revealed the existence of two key *cis*-acting elements i.e. TATA and CAAT boxes in the promoter and enhancer parts of all gene sequences. In addition, 5 classes of PM and DM-responsive regulatory elements were identified, namely defense and stress responsive element, salicylic acid responsive element, wound and pathogen responsive element, stress responsive element, As-1 and TGA element. Among all, the defense and stress responsive element was located in 12 PM-responsive and 10 DM-responsive promoter sequences, salicylic acid responsive element in 15 each PM-responsive and DM-responsive sequences, wound and pathogen responsive element in 17 PM-responsive and 16 DM-responsive sequences, As-1 in 10 each PM-responsive and DM-responsive sequences and TGA motif in 6 PM-responsive and 9 DM-responsive promoter sequences (Fig. 6). Taken together, the most frequent *cis* elements found in maximum number of PM and DM-responsive gene sequences were from wound and pathogen responsive element category.

## Discussion

As the cultivation of grapes is vulnerable to the emergence of diseases like PM and DM, efforts are increasingly directed towards the understanding of defense mechanisms of disease resistance at the molecular level. Various signaling pathways are triggered in plants at molecular level during pathogenic infection that provide plant defense. It has been reported in literature that during invasion by biotrophic organisms, the active defense response in plants is signaled by SA [32]. In the present study, we have identified various defense-responsive genes (*EDS1, NDR1, PAD4, NPR, RAR1* and *PR*) involved in SA signaling pathway. The members of these gene families have also been identified previously in grapevine and other plants, but there is no report of genome-wide recognition study of such gene families. The phylogenetic analysis of *V. vinifera* defensive gene families is conducted with model plant *A. thaliana* to understand their evolutionary relatedness. The findings show that the orthologous genes from both plants share a close evolutionary relatedness with each other, implying that the *EDS1, NDR1, PAD4, NPR, RAR* and *PR* genes have been conserved in different plants. The evolutionary conservation also indicated that these gene families play an important role in plant defense. Additionally, the paralogous defensive genes are found to be closely grouped, which indicated their structural homology.

Next, the changes in the expression patterns of above mentioned defensive gene families under pathogenic and non-pathogenic conditions are studied by conducting differential expression. Our analysis depicted that during infection, most of the defensive gene classes were up-regulated relative to non-pathogenic conditions, indicating the active involvement of SA-mediated defense pathway during infection with PM and DM. To confirm the TPM findings, we performed real time PCR of PM and DM infected leaf samples to test differences in relative expression between healthy and infected samples. In addition, since SA plays a prominent role in activating the defense mechanism, we gave SA treatment to leaf samples at different intervals of time. The results of real time study revealed that during SA treatment, PM infection and DM infection, most of the defense-responsive genes were up-regulated relative to control condition. This meant that anytime the plant suffers an outbreak, the innate immune response of the plant is activated through which various signaling pathways and comes into action. The expression level of defense-responsive genes is enhanced during infection as compared to non-pathogenic condition that eventually induces SAR in the plant. We found that in case of PM-responsive genes, initially, the expression level of most genes increase from 1 hpi to 2 hpi of SA treatment; however, a sudden decrease in expression levels was witnessed after 2 hpi till 2 dpi. After that, ultimately, at 3 dpi, a tremendous increase in most of the genes was visualized. In case of DM-responsive genes, initially, the defense-responsive genes were highly expressed at 1 hpi of SA treatment; after that there was a decline in expression levels; and finally at 3 dpi, most of the defensive genes were up-regulated. The possible assumption would be that following an infection, SA levels will rise in plant, leading to defense reaction. Thus, when we gave SA treatment to our samples, initially up-regulation of genes occurs at 1 h or 2 h; and later on to provide SAR to the plant, the expression levels of both PM and DM-responsive defensive genes peaks up at 3 dpi. Additionally, our results of powdery and downy mildew infection through quantitative real time PCR are in accordance with the findings of the TPM study. Most of the genes were up-regulated during PM and DM infections as compared to control conditions in both TPM and real time studies.

Past studies have shown that over-expression of these genes confers broad spectrum resistance to infected plants. For instance, one analysis in *Arabidopsis* showed that over-expression of *EDS1* enhances the accumulation of PR1 protein that ultimately provides broad spectrum resistance to the plant [41]. NPR proteins have been reported to engage in SA signal transduction in various plants. Once activated by SA, these genes regulate the expression of *PR* genes by interacting with various TFs. Through differential expression analysis, we have identified TFs of 3 different classes (bZIP, WRKY and NAC), that might activate PR proteins by interacting with NPR1 and NPR2 proteins. Previously, it has been shown in *Arabidopsis* that the *PR-1* gene expression is regulated through *NPR1* gene binding to TGA transcription factors [42]. Similarly WRKY and NAC transcription factors were also found to interact with *NPR1* gene in previous studies [43, 44]. Additionally, over-expression of the *NPR1* gene in plants like *Arabidopsis*, wheat, tomato and apple provides broad spectrum resistance against fungal and bacterial pathogens [45–48]. In one study, conducted in Fuji apples, it has been shown that over-expression of *NPR1* genes enhances PM resistance through SA mediated signaling pathway [49]. All these studies supported our results of expression analysis of various defense responsive genes conducted in grapes. Next, we performed the co-expression analysis of different genes and constructed their co-expression networks. Such networks provide very important information about candidate disease gene identification, regulatory genes identification, gene annotation etc. [50]. Apart from conventional co-expression networks, differential co-expression analysis is becoming more popular now-a-days [51]. This method identify genes with different co-expression pairs under diverse conditions like disease states, developmental stages, tissue forms etc. [51–56]. In the present study, we performed differential co-expression between the differentially expressed transcripts obtained from the gene expression data. All gene classes were analyzed for co-expression by taking two gene classes at a time. First, a pairwise correlation was examined for each gene class pair. Next, highly co-expressing transcripts with a threshold value of 0.99 to 1 were screened and their co-expression networks were constructed.

Next, to allocate roles to PM and DM-responsive defensive genes identified in the present research, Blast2GO was done. The study of GO annotations has showed that the maximum defense-responsive gene sequences are engaged in biological processes, accompanied by sequences in cellular component and molecular function category. The GO terms in biological process category are directly implicated in defense response which supported the functioning of proteins identified in our study in resistance mechanism. The proteins in cellular component class are found to perform their role inside cell which clearly indicated that the defensive pathway we are targeting is functional inside the cell. In the category of molecular processes, many of the proteins are involved in binding activity which supported the interacting nature of defense-responsive proteins.

In our research, we have recognized multiple regulatory elements that react to pathogenic stress, namely defense and stress responsive element (TC-rich repeats), salicylic acid responsive element (TCA element), wound and pathogen responsive element (WUN-motif and W-box), stress responsive element (GT1 Box), As-1 and TGA; they provide an important information regarding upstream control of PM and DM-responsive defensive proteins. The function of the TFs in response to PM and DM-stress has been well elucidated in previous studies. For example; the NAC TFs associate with GT1 box, bZIP bind to TGA elements and WRKY TFs associates with W-box in reaction to fungal infection [57–59]. In the present analysis, we have observed the involvement of these elements in defensive proteins, which clearly confirmed that these genes were up-regulated as a defense mechanism in the situation of PM and DM stress. Altogether, numerous regulatory elements are identified in the present study that could play an important role in regulating the expression of stress-responsive genes.

In the present study, we focused on molecular aspects and gene expression analysis to identify various defense-responsive genes and study their response against PM and DM infections in grapes. In future, the identified genes can be characterized for resistance against PM and DM in Arabidopsis plants. Further, the functionally validated genes will be available for transgenic studies in grapes for imparting fungal resistance.

## Conclusion

In our research, grapevine genome was examined to identify various classes of defensive genes that were directly implicated in *R-gene* mediated defense signaling during powdery and downy mildew infections. In total, we identified 6, 21, 2, 5, 3 and 48 *EDS1, NDR1, PAD4, NPR, RAR* and *PR* genes in the grape genome amongst which 27 PM-responsive and 59 DM-responsive differentially expressed defensive genes *(NLR, EDS1, NDR1, PAD4, NPR, RAR* and *PR*) were identified in 2 different *V. vinifera* accessions. The gene co-expression study depicted 24 PM-responsive and 22 DM-responsive defensive genes and Transcription factors (TF) that were found to be highly co-expressing with each other. Taken together, we can summarize that defensive genes identified in the current research could be beneficial in improving grapevine resistance to powdery and downy mildew.

## Methods

### Genome-wide identification of various defense-responsive gene families in *Vitis vinifera*

For the identification of *V. vinifera EDS1, NDR1, PAD4, NPR, RAR1* and *PR* gene families, all possible protein coding sequences were procured from NCBI genome database (ftp://ftp.ncbi.nlm.nih.gov/genomes/Vitis_vinifera/protein/) and constructed their local sequence database. Next, the previously documented protein sequences of above listed gene families from different plants namely *Arabidopsis thaliana, Arabidopis lyrata, Oryza sativa, Populus trichocarpa* and *Vitis vinifera* were obtained from NCBI (http://www.ncbi.nlm.nih.gov/protein) and their FASTA sequences were compiled into a file which was considered as a query file. To identify the putative hits in the grape genome, standalone protein BLAST (Basic Local Alignment Search Tool) was performed between the database and query file created above and the e-value used was 1e-05, respectively. The candidate gene sequences were verified by assessing the occurrence of respective domains of various gene families at different servers such as Grape Genome Browser (12X) (http://www.genoscope.cns.fr/externe/GenomeBrowser/Vitis/), conserved domains database (CDD) (http://www.ncbi.nlm.nih.gov/Structure/cdd/wrpsb.cgi) [60], InterProScan (https://www.ebi.ac.uk/interpro/search/sequence-search) [61] and SMART (http://smart.embl-heidelberg.de/) [62]. Next, the ‘In silico*’* characterization of defense-responsive gene families was done based on their gene structure, motif presence, chromosomal locations and physicochemical properties. For the gene structure prediction, an online tool ‘Splign’ was used to detect exons and introns (http://www.ncbi.nlm.nih.gov/sutils/splign/splign.cgi/). Visualization of exon and intron locations was done by using an online tool i.e. GSDS2.0 (http://gsds.cbi.pku.edu.cn/) [63]. The presence of conserved motifs was predicted through Multiple Expectation Maximization for Motif Elicitation (MEME) Suite (http://meme-suite.org/) and Pfam database (https://pfam.xfam.org/) [64, 65]. To determine the chromosomal locations, Grape Genome Browser at Genoscope (http://www.genoscope.cns.fr/externe/GenomeBrowser/Vitis/) and Ensembl Plants portal (https://plants.ensembl.org/Vitis_vinifera/Location/Genome/) were used. The physicochemical properties of defensive gene families were depicted by using ProtParam bioinformatics tool (https://web.expasy.org/protparam/) with default parameters [66].

### Multiple sequence alignment and phylogenetic analysis

Multiple sequence alignment of protein sequences of *EDS1, NDR1, PAD4, NPR, RAR1* and *PR* gene families was performed to identify conserved residues using Clustal W program at default parameters (http://www.ebi.ac.uk/Tools/msa/clustalw2/). To study the evolutionary relatedness of EDS1, NDR1, PAD4, NPR, RAR1 and PR proteins with *A. thaliana,* phylogenetic trees of all gene families were constructed following the Neighbor-joining method in MEGA 7 with 1000 bootstrap replicates. The JTT matrix-based method was used to compute the evolutionary distances [67–70]. For visualization of phylogenetic trees, an Interactive Tree of Life (iTOL) online software tool (https://itol.embl.de) was used that worked by taking Newick phylogenetic tree format as an input [71].

### Identification of PM and DM responsive defensive gene families in *V. vinifera*

To identify PM and DM responsive defensive genes, we made a FASTA file of protein coding sequences of defensive gene families *(EDS1, NDR1, NPR1, RAR1, PR)* and *NLR* genes identified in our previous research [40]. In addition, as transcription factors (TFs) are also presumed to participate in *R-gene* mediated defense pathway, various transcription factors (TFs) of *V. vinifera* belonging to 3 distinct families i.e. TGA, WRKY and NAC were retrieved from Plant Transcription Factor Database (TFDB) [72]. The RNA-seq data of two *V. vinifera* varieties i.e. Thompson Seedless and Pinot Noir was procured from NCBI’s Sequence Read Archive (SRA) database and the project I.Ds for these studies were SRP116308 and PRJEB24540 (http://www.ncbi.nlm.nih.gov/sra/). The transcriptomic data of Thompson Seedless was responsive to PM and was accessible at 1 time point i.e. 36 h post inoculation (hpi) (Northwest A&F University). The RNA-seq data of Pinot Noir was DM-responsive and retrieved at 3 time points i.e. 1 dpi, 2 dpi and 3 dpi [73]. Each of the derived SRA data consisted of three biological replicates. The identification of PM and DM-responsive defensive genes is based on the digital expression analysis of different groups of proteins identified in our study. The Trinity-V2.4 package’s RSEM (RNA-Seq by Expectation-Maximization) program was used to quantify the abundance of all PM and DM-responsive defensive genes as transcript per kilobase million (TPM) [74]. All three biological replicates of every condition were analyzed individually. Thereafter, EdgeR was used to calculate the differential gene expression by assigning a cut-off value of 4-fold change and *P*-value of 0.001. The visualization of differential gene expressions was done by constructing heat maps with Hierarchical Clustering Explorer 3.5 (http://www.cs.umd.edu/hcil/hce/) [75].

### Co-expression analysis of PM and DM responsive defensive genes in *V. vinifera*

Next, we conducted co-expression (CE) analysis of differentially expressed defensive genes to identify a group of genes expressing simultaneously during PM and DM-infection. The software used for detecting the co-expression was CoExpress 1.5.2. This is a stand-alone software tool that is focused on the Pearson correlation coefficient (R). The parameters were set to default while running this software. (http://Bioinformatics.lu/CoExpress/). The expression values of two sets of genes were loaded at a time and a linear correlation was measured amongst them. Finally, we screened genes that are highly co-expressing with the R value of 1 or close to 1. The visualization of co-expression networks for genes was achieved using the software Gephi 0.91 [76].

### Plant material and treatment

As SA is playing a pivotal role in *R-gene* mediated defense signaling against PM and DM-infections, we studied its effect on Thompson Seedless variety of *V. vinifera*. The stem cuttings were collected from National Research Centre for Grapes (NRCG), Pune. The Centre has the National Active Germplasm Site for grapes, where the grape germplasm is being maintained. The institute code number for Thompson seedless variety of *V. vinifera* is A37–3. The collected stem cuttings were grown in pots containing soil:soil-rite in the ratio of 2:1 in growth chamber in Department of Biotechnology, Panjab University, Chandigarh**.** The experiment was performed at 5 different time intervals i.e. 1 hpi, 2 hpi, 1 dpi, 2 dpi and 3 dpi in the sets of three biological replicates at each time point. The SA treatment was given at the final concentration of 100 mg/litre. The control and treated leaves were collected, snap frozen and stored in − 80 °C till further use. Additionally, we also studied the effects of PM and DM infection on the activity of defense-responsive genes identified in the present study. We collected the healthy and infected leaf samples of Thompson Seedless variety of *V. vinifera* from National Research Centre for Grapes (NRCG), Pune. The time points for PM and DM infected leaf samples collected were 2 dpi for PM infected samples and 1 dpi and 3 dpi for DM infected samples. During sample collection, the symptoms were not evident on the leaf surface. The collection was done on the basis of SRA data. During visible condition, the PM-infected leaf is covered with a dusty whitish powdery growth and DM appears as yellowish circular spots encircled by a brownish-yellow halo that covers the majority of the leaf surface. This experiment was also performed in the sets of three biological replicates at the respective time points.

### RNA isolation, cDNA synthesis and quantitative real time PCR analysis

Based on the genes identified in the co-expression analysis, we performed quantitative real time PCR (qPCR) of PM and DM-responsive differentially co-expressed defensive genes to measure the relative expression. Leaf samples were used to extract the total RNA by following Ghawana et al. 2011 protocol and the cDNA was prepared using the Superscript III first strand cDNA synthesis kit (Invitrogen USA) [77].

We used two housekeeping genes namely *actin (ACT)* and *elongation factor 1 (EF1)* as internal reference genes for the normalization of qPCR results. The real time primers were designed by using Primer 3 software (http://primer3.ut.ee/). The qPCR experiment was performed with three replicates of healthy, PM-infected, DM-infected and SA treated leaf samples by employing Bio-Rad CFX96 Real-Time PCR detection system. The following conditions were used while performing an experiment: 95 °C for 7 min, followed by 40 cycles of 95 °C for 20 s, Tm for 20 s and 72 °C for 20 s. In order to interpret the results of qPCR, we used REST 2009 algorithm (Qiagen) and 2(−∆∆CT) method to obtain relative gene expression ratios of target genes with respect to control genes (http://www.REST.de.com/) [78, 79]. All the triplicate experiments are represented as means ± standard deviations (SDs). Analysis of variance (ANOVA) followed by Tukey’s multiple comparisons test was conducted at *p*-value < 0.05 to detect the significant differences in gene expressions amongst control and treatment conditions using GraphPad Prism 9.2.0 software.

### Functional annotation of PM and DM responsive defensive genes in *V. vinifera*

For assigning biological functions to PM and DM-responsive defensive gene sequences, Blast2GO (https://www.blast2go.com/blast2go-pro/) tool was used [80]. Essentially, Blast2GO annotation requires 3 steps: BLAST to locate homologous sequences, Mapping to allocate GO terms to each hit obtained, Annotation to assign role to query sequences on the basis of their cellular positions, molecular functions and biological processes. Furthermore, knowledge regarding various protein domains or motifs may also be obtained from Blast2GO by running InterProScan.

### Promoter study of PM and DM responsive defensive genes in *V. vinifera*

We also predicted various *cis*-acting regulatory elements of PM and DM-responsive defensive genes by using PlantCare database with default parameters (http://bioinformatics.psb.ugent.be/webtools/plantcare/html/) [81]. To conduct the analysis, an upstream sequence from transcription start site was needed. Nucleotide BLAST was then done between genomic sequence and coding sequence of respective gene. Eventually, different regulatory elements were predicted that might be playing an important role in the regulation of PM and DM-responsive genes of defense pathway.

## Supplementary Information

**Additional file 1 **Protein IDs, chromosomal locations and physicochemical properties of *EDS1* (Sheet 1), *NDR1* (Sheet 1), *PAD4* (Sheet 1), *NPR* (Sheet 1), *RAR1* (Sheet 1) and *PR* (Sheet 1) defensive genes. The table describes the protein ID, transcript ID, chromosomal position, polypeptide length, molecular weight, isoelectric point and instability index of various defensive gene families.

**Additional file 2.** Gene configuration and motif study of various classes of defensive genes. The arrangement of introns and exons is defined by GSDS 2.0 server and motif analysis was conducted with Pfam database and MEME tool (A) Gene structure and conserved domains of EDS1 class of defensive genes. (B) Gene structure and conserved domains of NDR1 class of defensive genes. (C) Gene structure and conserved domains of NPR class of defensive genes. (D) Gene structure and conserved domains of PAD4 class of defensive genes. (E) Gene structure and conserved domains of PR class of defensive genes. (F) Gene structure and conserved domains of RAR1 class of defensive genes. Exons are represented by yellow boxes partitioned by thin intron lines and blue boxes reflect UTRs. Different types of conserved motifs are shown with different colored boxes.

**Additional file 3.** List of primers used for qRT-PCR analysis of selected PM and DM-responsive defensive genes as well as Internal control genes.

**Additional file 4.** Functional characterization of defensive genes by assigning Gene Ontology (GO) terms using Blast2GO tool. (a) Representation of (a) PM-responsive (b) DM-responsive defensive genes classified on the basis of GO terms enrichment in biological process, cellular component and molecular function categories.

## Data Availability

Not applicable.

## Abbreviations

ACT: Actin
CARE: Cis-acting Regulatory Elements
CE: Co-Expression
CNL: coiled-coil NLRs
DEA: Differential Expression Analysis
DM: Downy Mildew
DPI: days post inoculation
EDS1: Enhanced Disease Susceptibility
EF1: Elongation Factor 1
ETI: Effector Triggered Immunity
ET: Ethylene
TPM: Transcript per kilobase million
GO: Gene Ontology
GSDS: Gene Structure Display Server
HPI: hours post inoculation
HR: Hypersensitive Response
JA: Jasmonate
LEA2: Late embryogenesis abundant
MAPK: Mitogen activated protein kinase
MEME: Multiple Expectation Maximization for Motif Elicitation
NLR: Nucleotide-Binding Leucine Rich Repeat Receptor
NDR1: Non-Race-specific Disease Resistance
NPR: Nonexpressor of PR
nr: Non Redundant
PAD4: Phytoalexin-deficient 4
PAMP: Pathogen-associated Molecular Patterns
PBS2: avrPphB susceptible 2
pI: Isoelectric point
PM: Powdery Mildew
PR: Pathogenesis Related
PRR: Pattern Recognition Receptors
PTI: Pathogen Triggered Immunity
qPCR: quantitative real-time PCR
R: Resistance
RAR1: Required for Mla-specified Resistance
RSEM: RNA-Seq by Expectation-Maximization
SA: Salicylic Acid
SAG101: Senescence associated gene 101
SAR: Systemic Acquired Resistance
SRA: Sequence Read Archive
TF: Transcription Factor
TNL: TIR-type NLRs
