# Supplementary figures and images for: Identification of defense related gene families and their response against powdery and downy mildew infections in Vitis vinifera
Source: BMC Genomics. 2021 Oct 30;22:776. doi: 10.1186/s12864-021-08081-4 (PMC8556916; doi:10.1186/s12864-021-08081-4)

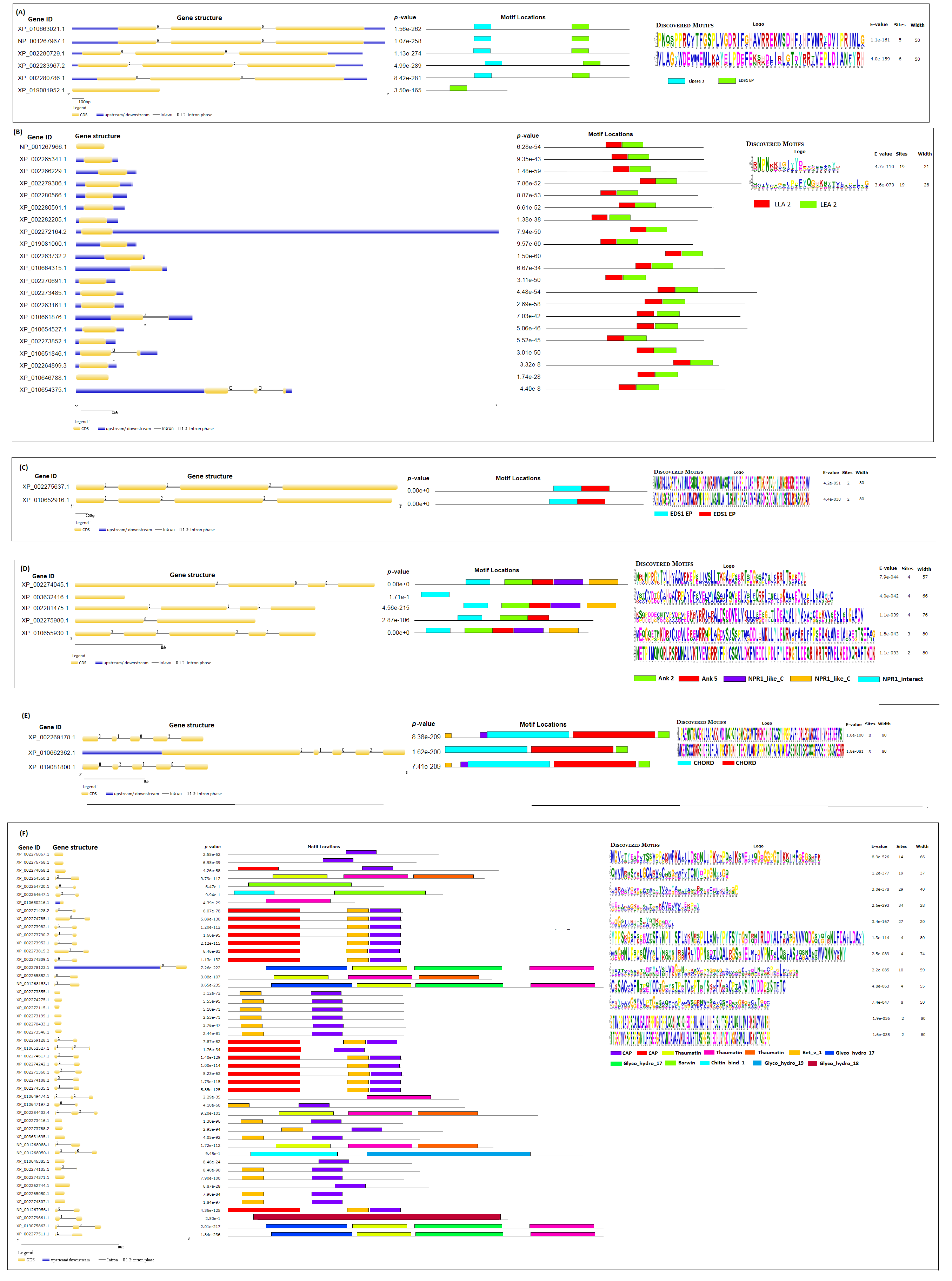

Supplement: Supplementary file 2 — Additional file 2. Gene configuration and motif study of various classes of defensive genes. The arrangement of introns and exons is defined by GSDS 2.0 server and motif analysis was conducted with Pfam database and MEME tool (A) Gene structure and conserved domains of EDS1 class of defensive genes. (B) Gene structure and conserved domains of NDR1 class of defensive genes. (C) Gene structure and conserved domains of NPR class of defensive genes. (D) Gene structure and conserved domains of PAD4 class of defensive genes. (E) Gene structure and conserved domains of PR class of defensive genes. (F) Gene structure and conserved domains of RAR1 class of defensive genes. Exons are represented by yellow boxes partitioned by thin intron lines and blue boxes reflect UTRs. Different types of conserved motifs are shown with different colored boxes. [file 12864_2021_8081_MOESM2_ESM.png]

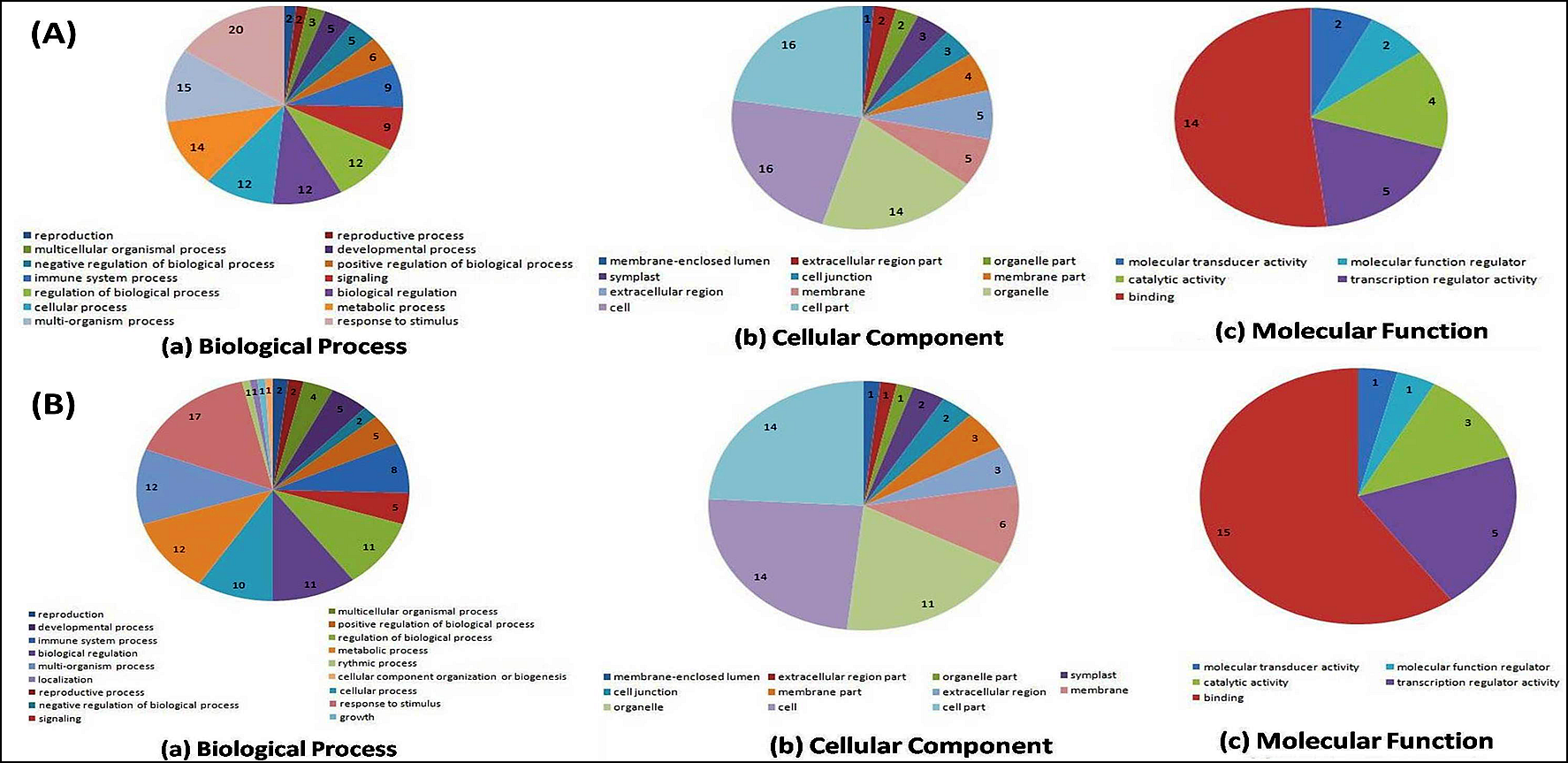

Supplement: Supplementary file 4 — Additional file 4. Functional characterization of defensive genes by assigning Gene Ontology (GO) terms using Blast2GO tool. (a) Representation of (a) PM-responsive (b) DM-responsive defensive genes classified on the basis of GO terms enrichment in biological process, cellular component and molecular function categories. [file 12864_2021_8081_MOESM4_ESM.png]
