# Supplementary material for: Identification of defense related gene families and their response against powdery and downy mildew infections in Vitis vinifera
Source: BMC Genomics. 2021 Oct 30;22:776. doi: 10.1186/s12864-021-08081-4 (PMC8556916; doi:10.1186/s12864-021-08081-4)
Supplement: Supplementary file 3 — Additional file 3. List of primers used for qRT-PCR analysis of selected PM and DM-responsive defensive genes as well as Internal control genes. [file 12864_2021_8081_MOESM3_ESM.docx]

**Table 1: List of primers used for qRT-PCR analysis of PM-responsive defensive genes as well as Internal control genes**

| **_Gene ID_** | **_Primer name_** | **_Primer sequence_** | **_Amplicon length (bp)_** | **_Amplicon Tm_** |
| --- | --- | --- | --- | --- |
| XP_002279295.1 | XP_002279295.1 F  XP_002279295.1 R | **5’-**CTGATGCAGTCAAGGAGATACCTA**-3’**  **5’-**GGATCTTATCAGCAACAAAGTCCT**-3’** | 237 | 55 |
| XP_010660247.1 | XP_010660247.1 F  XP_010660247.1 R | **5’-**CCTCATCGATTTGTAGTCAGAGAA**-3’**  **5’-**GTTGAAGGTAGTGTTGTGAAATGC**-3’** | 242 | 55 |
| XP_010661280.1 | XP_010661280.1 F  XP_010661280.1 R | **5’-**AGAGATTGGTCTTTATGCTGATCC**-3’**  **5’-**CGCTATTTCTTCTCCTCTTCTTTG**-3’** | 223 | 55 |
| XP_010650651.1 | XP_010650651.1 F  XP_010650651.1 R | **5’-**TGGTCCACTGTACTTCAACAGTTT**-3’**  **5’-**AACGTCTTCTGGAGTTTCATTCTC**-3’** | 222 | 55 |
| XP_010650653.1 | XP_010650653.1 F  XP_010650653.1 R | **5’-**GCAGTCAACTCACAAGGTCAAAGT**-3’**  **5’-**AATTTGCTAGGGTTGTCTTTCCAG**-3’** | 214 | 55 |
| NP_001267967.1 | NP_001267967.1 F  NP_001267967.1 R | **5’-**GGAGAAACAAAGATGGATGCTAGT**-3’**  **5’-**AGAAGTATAGAGTTGCCAGGATGG**-3’** | 223 | 53 |
| XP_002281475.1 | XP_002281475.1 F  XP_002281475.1 R | **5’-**TCCCTAGATGCTCAGAAGTACTCA**-3’**  **5’-**CTAGAGATGTGGATGATGATGAGG**-3’** | 210 | 53 |
| NP_001267966.1 | NP_001267966.1 F  NP_001267966.1 R | **5’-**TCTGGCTTTACCTTTCTGTTCTCT**-3’**  **5’-**CCCTTGTAAGAGGCATAAACTTGT**-3’** | 230 | 53 |
| XP_002284403.4 | XP_002284403.4 F  XP_002284403.4 R | **5’-**GATGGCTTCAACCTACCATTAGTT**-3’**  **5’-**CTTGAACTTCTTGGAGTAGGAGGT**-3’** | 240 | 53 |
| XP_002273790.2 | XP_002273790.2 F  XP_002273790.2 R | **5’-**AGCTGAACTTTGCTGAAGGTAGCC**-3’**  **5’-**GGGTTTGGAATGATACACGCTCAC**-3’** | 209 | 53 |
| XP_002274275.1 | XP_002274275.1 F  XP_002274275.1 R | **5’-**CTACCTACGCCCAGAACTATGCTA**-3’**  **5’-**CACTGAGTTGCTCCAAACAACCTG**-3’** | 224 | 53 |
| XP_002276867.1 | XP_002276867.1 F  XP_002276867.1 R | **5’-**GAGACCATATACCGGGTTTCCAAG**-3’**  **5’-**CATGTATCGCCGTTACCCCAGTAT**-3’** | 235 | 53 |
| NP_001268153.1 | NP_001268153.1 F  NP_001268153.1 R | **5’-**TGAGACTCTACGACCCTAACCAAG**-3’**  **5’-**CCCGTAGGGCTTACTTCATTTCC**-3’** | 205 | 53 |
| XP_002281328.1 | XP_002281328.1 F  XP_002281328.1 R | **5’-**ACTGGTAATATGGATCCTGCTGAT**-3’**  **5’-**CACCTTTGTTCTCAATGTCTCAAC**-3’** | 246 | 53 |
| Vv Actin | ACT F  ACT R | 5’- GGTGATGATGCTCCCAGGGC-3’  5’- ACTGGGTGTTCTTCAGGGGC-3’ | 242 | 57 |
| Vv EF1 | EF1 F  EF1 R | 5’- TGGTGTCCTGAAGCCCGGTA-3’  5’- TGGGAGGTGAAGTTGGCTGC-3’ | 222 | 57 |

**Table 2: List of primers used for qRT-PCR analysis of DM-responsive defensive genes as well as Internal control genes**

| **_Gene ID_** | **_Primer name_** | **_Primer sequence_** | **_Amplicon length (bp)_** | **_Amplicon Tm_** |
| --- | --- | --- | --- | --- |
| XP_003633380.1 | XP_003633380.1 F  XP_003633380.1 R | **5’-**GATACATCAAACAGGAGCAGAATG**-3’**  **5’-**CTGTCAATTTTACGATCAGGAGTG**-3’** | 229 | 55 |
| XP_010650653.1 | XP_010650653.1 F  XP_010650653.1 R | **5’-**GATGGCCTAGACAGGTTACTCAAT**-3’**  **5’-**ACCTCCCAAGCAAGTAAATGTAAG**-3’** | 229 | 55 |
| XP_003634660.1 | XP_003634660.1 F  XP_003634660.1 R | **5’-**GTTCAGCAGTATCATCGTTTTCTG**-3’**  **5’-**CTCACTTTACAGCCAGTTTCTTCA**-3’** | 214 | 55 |
| XP_010645044.1 | XP_010645044.1 F  XP_010645044.1 R | **5’-**TGGTCCACTGTACTTCAACAGTTT**-3’**  **5’-**AACGTCTTCTGGAGTTTCATTCTC**-3’** | 222 | 55 |
| XP_002265341.1 | XP_002265341.1 F  XP_002265341.1 R | **5’-**TTACCAGGAGCCGAAGAACACAAC**-3’**  **5’-**TAAGCACCCAAAATGTTTCCATCC**-3’** | 240 | 53 |
| XP_010654527.1 | XP_010654527.1 F  XP_010654527.1 R | **5’-**ACCTCTCTCCGAATCTCCCAATTC**-3’**  **5’-**CTGTCGAGGGTTTCTGAAGTGGAT**-3’** | 248 | 57 |
| XP_002280786.1 | XP_002280786.1 F  XP_002280786.1 R | **5’-**GAGAAGAAGCGAGTTATTTTCACC**-3’**  **5’-**GGGATCACATCGTATCTCATAACA**-3’** | 221 | 57 |
| XP_019081952.1 | XP_019081952.1 F  XP_019081952.1 R | **5’-**TGTTTCTGGGCTGAGGTAGAGGAC**-3’**  **5’-**ATGCGAGATGATTTGGGTTCTGAT**-3’** | 218 | 54 |
| XP_002284403.4 | XP_002284403.4 F  XP_002284403.4 R | **5’-**GCTCATGCATCAGAAAGGACTTCA**-3’**  **5’-**CAGCCATTGTTGGTGAAGAGATTG**-3’** | 202 | 56 |
| XP_002273790.2 | XP_002273790.2 F  XP_002273790.2 R | **5’-**ATCGAAACAGTGGAAGGAAATGGA**-3’**  **5’-**CAGGACTGGCCTCAAATGAAATCT**-3’** | 205 | 53 |
| XP_002276768.1 | XP_002276768.1 F  XP_002276768.1 R | **5’-**AATGGGCAGTACGGAGAGAACATC**-3’**  **5’-**TTGCAAGTGATGAAGACACCCCTA**-3’** | 230 | 54 |
| XP_010662362.1 | XP_010662362.1 F  XP_010662362.1 R | **5’-**CTACTTCAGTTACACTGGCGACAC**-3’**  **5’-**TTGTAGTAAGTTCTGGCATTGTCC**-3’** | 246 | 55 |
| XP_010663931.1 | XP_010663931.1 F  XP_010663931.1 R | **5’-**CTCAAGTAGTGCTACTCCAACGAA**-3’**  **5’-**TGAGTCATCCCTATCCCTTTCTAC**-3’** | 238 | 55 |
| Vv Actin | ACT F  ACT R | 5’- GGTGATGATGCTCCCAGGGC-3’  5’- ACTGGGTGTTCTTCAGGGGC-3’ | 242 | 57 |
| Vv EF1 | EF1 F  EF1 R | 5’- TGGTGTCCTGAAGCCCGGTA-3’  5’- TGGGAGGTGAAGTTGGCTGC-3’ | 222 | 57 |
